# Status of Hypertension in Tehran: Potential impact of the ACC/AHA 2017 and JNC7 Guidelines, 2012–2015

**DOI:** 10.1038/s41598-019-42809-3

**Published:** 2019-04-23

**Authors:** Samaneh Asgari, Pegah Khaloo, Davood Khalili, Fereidoun Azizi, Farzad Hadaegh

**Affiliations:** 1 Prevention of Metabolic Disorders Research Center, Research Institute for Endocrine Sciences, Shahid Beheshti University of Medical Sciences, Tehran, Iran; 2 Endocrinology and Metabolism Research Center (EMRC), Vali-Asr Hospital, School of Medicine, Tehran University of Medical Sciences, Tehran, Iran; 3 Endocrine Research Center, Research Institute for Endocrine Sciences, Shahid Beheshti University of Medical Sciences, Tehran, Iran

**Keywords:** Population screening, Epidemiology

## Abstract

This study aimed to determine the prevalence of hypertension, the recommended anti-hypertensive therapy and the percentage of hypertensive patients who had achieved the blood pressure (BP) target according to 2017 American College of Cardiology/American Heart Association (ACC/AHA) versus JNC7 and 8 guidelines, among Iranian population. Data of participants aged ≥20 years from the fifth phase (2012–2015) of the Tehran lipid and glucose study (N = 10,576) were analyzed, using survey analysis. The weighted prevalence of hypertension among those not on anti-hypertensive medications was 42.7 and 12.6%, applying the ACC/AHA and JNC7 guideline definitions, respectively; the corresponding values with including BP-lowering medication in definition of hypertension were 47.1% and 20.4%, respectively. However, 90% of these hypertensive people were found to have a 10-year cardiovascular disease risk of <10%. Applying the ACC/AHA guideline, anti-hypertensive medication was recommended for 21.9% of Tehranians, compared to 19.3 and 12.2% according to the JNC7 and 8 guidelines, respectively. Among Tehranians taking anti-hypertensive medication, 20% achieved the BP goal according to the ACC/AHA guideline, compared to the 42.1 and 53.6%, using JNC7 and 8 guidelines, respectively. Despite the tremendous increase in the prevalence of hypertension, most of the newly identified cases did not belong to the high-risk group.

## Introduction

Hypertension is the strongest single contributor to the global burden of disease and all-cause mortality worldwide^1^, being responsible for 9.4 million deaths in 2010. Increased blood pressure contributes to cardiovascular and cerebrovascular events including stroke, dementia and myocardial infarction (MI)^2^. Findings from serial surveys show an increasing prevalence of hypertension in developing countries, particularly in urban areas^3^. Most of the disease burden caused by high blood pressure is attributed to low and middle-income countries^4^. A recent study reported a high prevalence of non-communicable disease (NCD) risk factors including hypertension, diabetes, dyslipidemia and obesity in the Middle East and North Africa (MENA) countries^5^. In 2005, high blood pressure was responsible for 80,000 deaths in Iran^6^. Moreover, we previously reported that increased risk of cardiovascular disease (CVD) and all-cause mortality events are related to hypertension in middle-aged and elderly Iranian populations^7^; in fact over 20 and 17% of CVD and all-cause mortality have been attributed to this risk factor^8^.

The 2017 guideline for high blood pressure of the American College of Cardiology/American Heart Association (ACC/AHA), provides comprehensive information on the prevention, management, and treatment of hypertension^9^; this guideline updated the 2003 Seventh Report of the Joint National Committee^10^ (JNC7) and the 2014 eight-panel member report (JNC8) guideline^11^ and documented a new definition for hypertension and blood pressure target goals. The 2017 ACC/AHA guideline suggests lower systolic and diastolic blood pressure for the definition of hypertension (130/80 mmHg vs. 140/90 mmHg, respectively), compared to the 2003 JNC7. Additionally, the 2017 AHA/ACC guideline recommended antihypertensive medication at the level of systolic/diastolic blood pressure (SBP/DBP) 130/80 mmHg, for both high cardiovascular risk groups as well as elderly populations, aged ≥65 years, an issue not addressed in previous guidelines. There was a 13.7% increase in the prevalence of hypertension from 31.9 to 45.6%, in America when defined by the 2017 ACC/AHA guideline, compared with 2003 JNC7^12^.

The prevalence of hypertension and pre-hypertension, using 2003 JNC7 criteria, was reported to be 25.6 and 39.8% in Iranian adults in 2011^13^. Elsewhere in rural areas of Iran, this number was reported to be 42.7%^14^. It is believed that the 2017 ACC/AHA guideline has the potential to increase hypertension prevalence and use of anti-hypertensive medication^12^.

The purpose of this study is to determine the prevalence of hypertension, the recommended anti-hypertensive therapy and the percentage of hypertensive patients who had achieved the blood pressure (BP) target goal according to 2017 ACC/AHA guideline, compared to the 2003 JNC7, using the Munter *et al*.^12^ approach, among a sample of Tehranians. As acknowledged by Whelton PK, *et al*.^9^ “*The recommended BP classification system is most valuable in untreated adults as an aid in decisions about prevention or treatment of high BP”*(2017 High Blood Pressure Clinical Practice Guideline, Recommendation-Specific Supportive Text, page 21)”. Therefore, to report the prevalence, we focused mainly on untreated anti-hypertensive individuals. We also compared the prevalence of adults recommended anti-hypertensive medication and the percentage of patients who had achieved the blood pressure target goal according to the 2017 ACC/AHA vs. 2014 JNC8 guidelines.

## Materials and Methods

### Study population

The study sample was selected among participants of the Tehran Lipid and Glucose Study (TLGS), a population-based prospective study conducted on a representative sample of Tehranians (at baseline), to determine the risk factors for NCD and assess the prevention strategies for improving lifestyles. Data enrollment was done in two phases, i.e. the first (1999–2001; n = 15005) and the second (2002–2005; n = 3555). Data collection is ongoing and planned to continue for at least 20 years, at approximately 3-year intervals with prospective follow-ups; third phase: 2005–2008, fourth phase: 2009–2011 and fifth phase: 2012–2015. Details of sampling and study methods have been published elsewhere^15^.

Our study sample was derived from 10,721 individuals aged ≥20 years, who had participated in the fifth phase of the TLGS. Furthermore, we excluded participants with missing data on SBP and DBP (n = 103) and the covariates included in 10-year predicted CVD risk factors according to the Pooled Cohort risk equations^16^, i.e. fasting plasma glucose (FPG), high density lipoprotein cholesterol (HDL-C), total cholesterol (TC) and smoking (n = 42). Therefore, data of 10,576 participants were eventually available for the current study. The medical ethics committee of the Research Institute for Endocrine Sciences (RIES) approved the study proposal and experimental protocols. Written informed consent was obtained from all participants. All methods of the current study were performed in accordance with the relevant guidelines and regulations.

### Anthropometrics and laboratory measurements

Information on demographic data, personal and familial history of CVD, medical and medication history, and level of smoking habits were obtained by a trained interviewer using a standard questionnaire.

Based on the TLGS design^17^, using the Monica manual (part III, section I)^18^, two measurements of SBP and DBP were taken on the right arm after a 15-min rest in a sitting position. A Richter sphygmomanometer (Germany), the reliability and validity of which are confirmed and calibrated annually by the Institute of Standards & Industrial Research of Iran was used. The researcher was blinded to the samples, group (test or control). Furthermore, quality control was adopted to assure that data are collected uniformly over time according to the ARIC manuals^19^; the mean of two measurements was considered as the subject’s blood pressure.

A blood sample was taken after 12 to 14 hours overnight fasting between 7:00 and 9:00 AM from all study participants; FPG was measured using an enzymatic colorimetric method with glucose oxidase; inter- and intra-assay coefficients of variation (CV) at baseline and follow-up phases were both <2.3%. HDL-C was measured after precipitation of the apolipoprotein B containing lipoproteins with phosphotungistic acid. TC was assayed using the enzymatic colorimetric method with cholesterol esterase and cholesterol oxidase; both inter-and intra-assay coefficients of variation at baseline and follow-up phases were 1.9%. Serum creatinine (cr) level assessments were done using the kinetic colorimetric Jaffe.

All blood analyses were carried out in the TLGS research laboratory on the day of blood collection and analyses were performed using Pars Azmon kits (Pars Azmon Inc., Tehran, Iran) and a Selectra 2 auto-analyzer (Vital Scientific, Spankeren, Netherlands). Samples were only analyzed when internal quality control met the acceptable criteria.

### Definition of terms

**Figure 1:**
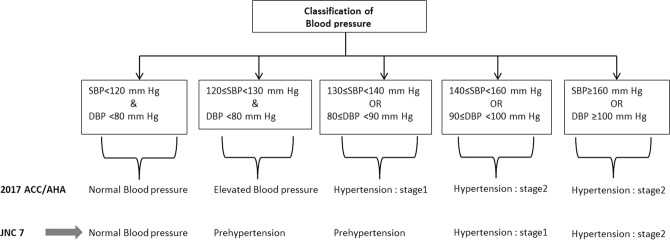
Classification of blood pressure according to the 2017 ACC/AHA and JNC7 guideline. 2017 ACC/AHA guideline: 2017 American College of Cardiology/American Heart Association Guideline for the Prevention, Detection, Evaluation, and Management of High Blood Pressure in Adults. JNC7 guideline: The Seventh Reportof the Joint National Committee on Prevention, Detection, Evaluation, and Treatment of High Blood Pressure. SBP: systolic blood pressure; DBP: diastolic blood pressure.

The definition of hypertension, criteria to recommend anti-hypertensive medication and treatment goal according to the 2017 ACC/AHA, 2003 JNC 7 and 2014 JNC8 are illustrated in Table 1. According to the 2017 ACC/AHA guideline, blood pressure among those not taking anti-hypertensive medication was categorized into five groups: Normal blood pressure (SBP < 120 and DBP <80 mmHg); elevated blood pressure (120–129 & <80 mm Hg); stage 1 hypertension (130–139/80–89 mm Hg), and stage 2 hypertension (≥140/≥90 mm Hg). Participants who were taking anti-hypertensive medication were categorized separately (Fig. 1). Smoking status was categorized into three categories, including current smokers, former smokers (those who used to smoke in the past) and never smokers. Estimated Glomerular Filtration Rate (eGFR; mL/min per 1.73 m^2^) was estimated by the Chronic Kidney Disease Epidemiology Collaboration (CKD‐EPI) abbreviated prediction equation^20^. CKD was defined as eGFR <60 mL/min per 1.73 m^2^ for >3 months^21^.

**Table 1:** Blood pressure levels used for hypertension definition, recommended anti-hypertensive medication and treatment goal according to the 2017 ACC/AHA guideline, the JNC7 guideline and the JNC8 panel member report.

	Definition of hypertension		
	2017 ACC/AHA	JNC7	JNC8^a^
General population			
SBP	≥130	≥140	≥140
DBP	≥80	≥90	≥90
**Recommended anti-hypertensive medication**			
General population			
SBP	≥140	≥140	≥140
DBP	≥90	≥90	≥90
Diabetes or CKD			
SBP	≥130	≥130	≥140
DBP	≥80	≥80	≥90
High cardiovascular disease risk			
SBP	≥130	—	—
DBP	≥80	—	—
Age ≥65 years			
SBP	≥130	—	—
DBP	≥80	—	—
Age ≥60 years without diabetes or CKD			
SBP	—	—	≥150
DBP	—	—	≥90
**Treatment goal among those on anti-hypertensive medication** ^**b**^			
General population			
SBP	<130	<140	<140
DBP	<80	<90	<90
Diabetes or CKD			
SBP	<130	<130	<140
DBP	<80	<80	<90
High cardiovascular disease risk^c^			
SBP	<130	—	—
DBP	<80	—	—
Age ≥65 years			
SBP	<130	—	—
DBP	<80	—	—
Age ≥60 years without diabetes or CKD			
SBP	—	—	<150
DBP	—	—	<90

- The same approach as other thresholds should be applied.

^a^Definitions of hypertension and prehypertension not addressed in JNC8; the same approach as JNC7 should be applied.

^b^To achieve treatment goal, both SBP and DBP goals have to be met.

^c^High cardiovascular disease risk is defined as 10-year predicted cardiovascular risk ≥10% using pooled cohort risk equation or prevalent of CVD.

2017ACC/AHA guideline: 2017 American College of Cardiology/American Heart Association Guideline for the Prevention, Detection, Evaluation, and Management of High Blood Pressure in Adults.

JNC7 guideline: The Seventh Report. Of the Joint National Committee on Prevention, Detection, Evaluation, and Treatment of High Blood Pressure.

JNC8 guideline: Panel member report- 2014 Evidence-Based Guideline for the Management of High Blood Pressure in Adults.

SBP: systolic blood pressure; DBP: diastolic blood pressure; CKD: chronic kidney disease;

### Statistical analysis

A survey analysis was conducted to generalize TLGS results to the adult Tehranian population; data were weighted directly to the 2016 urban population of Tehran^22^, based on the 2016 national Iranian census, to match the age (10-year strata) and gender strata.

Using 2017 ACC/AHA and 2003 JNC7 guidelines, the demographic and clinical characteristics of the TLGS population across blood pressure stages were reported using mean (SE) values for continuous and frequencies (%) for categorical variables. The weighted prevalence and the frequency of hypertension and recommended anti-hypertensive medications for TLGS individuals according to 2017 ACC/AHA and the 2003 JNC7 guidelines and those only included in the 2017 ACC/AHA guideline were calculated for the overall population according to the age strata, gender, 10-year risk categories and those with prevalent CVD. Moreover, the frequency and the percentage of drug-treated hypertensive TLGS individuals who had reached the blood pressure target goal, according to 2017 ACC/AHA and 2003 JNC 7 guidelines and those only included in the 2003 JNC7 guideline were calculated for the overall population according to the age strata, gender and different medication categories. Furthermore, sensitivity analysis was done by recalculating the prevalence of hypertension and the prevalence of adults recommended anti-hypertensive medication, including those treated with anti-hypertensive medication as prevalent hypertensive cases. The same approach was also applied for 2014 JNC8 panel member report. Statistical analyses were done using Stata version 12.0 (Stata Corp LP, College Station, Texas).

## Results

### Characteristics of TLGS data over hypertension categories

Applying the 2017 ACC/AHA guideline, the prevalence of different categories of blood pressure among Tehranian adults for normal blood pressure, elevated blood pressure and stages 1 and 2 hypertension were 48.0, 5.0, 26.6 and 10.0%, respectively. Additionally, 10.4% of adults were taking antihypertensive medication. Tehranian participants with higher blood pressure were older, had higher levels of TC, FPG and mean 10-year predicted CVD risk scores and lower levels of HDL-C (Table 2).

**Table 2:** Weighted characteristics of adults by blood pressure levels and anti-hypertensive medication according to the 2017 ACC/AHA guideline based on the 2012–2015 examination of TLGS data.

	SBP/DBP (mm Hg) categories among participants without anti-hypertensive medication^c^				Taking anti-hypertensive medication (n = 1580)
	Normal blood pressure (n = 4572)	Elevated blood pressure (n = 542)	Stage 1 hypertension (n = 2712)	Stage 2 hypertension (n = 1170)	
Percentage of Tehranian adults	48.0	5.0	26.6	10.0	10.4
Age, (years)	42.3	42.5	42.6	43.0	43.5
Female gender^a^, (%)	50.2	50.2	50.2	50.2	50.2
Current smoking, (%)	28.6	23.7	24.2	25.7	23.6
SBP, (mm Hg)	104.1 (0.13)	123.0 (0.14)	117.9 (0.19)	134.5 (0.52)	129.7 (0.97)
DBP, (mm Hg)	69.5 (0.1)	73.2 (0.3)	81.7 (0.1)	92.8 (0.3)	87.7 (0.7)
TC, (mmol/L)	4.8 (0.01)	4.9 (0.05)	5.0 (0.02)	5.1 (0.04)	4.9 (0.06)
HDL-C, (mmol/L)	1.3 (0.01)	1.2 (0.01)	1.2 (0.01)	1.2 (0.01)	1.3 (0.02)
FPG, (mmol/L)	5.3 (0.02)	5.5 (0.05)	5.5 (0.03)	5.7 (0.05)	6.0 (0.09)
eGFR, (ml/min/1.73 m^2^)	75.7 (0.2)	76.5 (0.6)	75.0 (0.2)	75.1 (0.5)	67.6 (0.8)
Mean 10-year predicted CVD risk^b^	2.4 (0.05)	3.0 (0.08)	2.9 (0.05)	3.9 (0.09)	4.2 (0.10)
Lipid lowering medication, (%)	6.3	6.7	5.9	5.2	21.6
Diabetes lowering medication, (%)	4.8	4.3	5.7	6.4	14.3
Diabetes, (%)	6.3	6.7	8.5	11.1	17.2
CKD, (%)	12.3	13.5	14.5	13.1	33.2
Prevalent CVD, (%)	5.4	4.2	4.5	5.0	27.1
10-year risk categories					
<5%	86.3	82.8	84.1	80.9	81.7
5%-<10%	6.3	7.3	7.0	7.0	5.3
10%-<20%	5.1	6.2	5.1	6.1	6.6
≥20%	2.3	3.6	3.8	6.0	6.4

Mean (SE) for continuous variable and weighted % were reported for categorical variables.

Crude sample size was reported for each blood pressure levels.

^a^Within category proportion (column %) of female were reported after age and gender adjustment; however, between category proportion (row %) are 28%, 1.92%, 10.7%, 3.62% and 6.14% for classification of hypertension; normal blood pressure, elevated blood pressure, stage 1 hypertension, stage 2 hypertension and those on medication, respectively.

^b^10-year predicted risk was calculated based on ACC/AHA pooled cohort risk equation^16^.

^c^According to the JNC 7 guideline, categories are defined as (Normal, pre-hypertension, prehypertension and stage 1 hypertension, respectively)

SBP: Systolic blood pressure; DBP: Diastolic blood pressure; TC: Total cholesterol; HDL-C: High density lipoprotein cholesterol; FPG: Fasting plasma glucose; eGFR: estimated glomerular filtration rate; CKD: chronic kidney dieses; CVD: Cardiovascular disease.

According to Sup Table 1, compared with the 2003 JNC7 guideline hypertension definition, Tehranian adults included only in the 2017 ACC/AHA guideline were younger, generally had more favorable CVD risk factors and ACC/AHA pooled risk scores, but showed higher rates of lipid lowering usage and had higher prevalence of CKD. Also, Tehranian adults, who were recommended anti-hypertensive medication, assessed only by the 2017 ACC/AHA guideline, were older, had more favorable CVD risk factors, higher value of eGFR and lower level of ACC/AHA pooled risk scores, compared with JNC7 guideline; however, they reported higher prevalence of CVD (76.3 vs. 4.8%, respectively).

Weighted characteristics of Tehranian adults taking anti-hypertensive medication with blood pressure, and those achieved the BP target goal are presented in Sup Table 2.

### Comparison of the 2017 ACC/AHA guideline with JNC7 guidelines

The percentage (95% CI) of Tehranian adults meeting the definition of hypertension and recommended anti-hypertensive medication, among those without anti-hypertensive medication, according to the 2017 ACC/AHA and the 2003 JNC7 guidelines based on the TLGS data are shown in Table 3. Accordingly, the prevalence of hypertension was 42.7% and 12.6% using the 2017 ACC/AHA and the 2003 JNC7 guidelines definitions, respectively; higher prevalence of hypertension with the updated definition was observed, regardless of age groups, gender, presence of prevalent CVD and 10-year CVD risk categories. Accordingly, the greatest difference was seen in individuals aged 40–60 years, male gender and those who had a 10-year predicted risk of 5–10%. Using the 2017 ACC/AHA guideline, anti-hypertensive medication was recommended for 21.9% of Tehranian adults, compared to 19.3%, according to the 2003 JNC7 guideline; the higher rates were present regardless of age groups, gender, the presence of prevalent CVD and 10-year CVD risk categories. The greatest difference in the prevalence of adults recommended medication was observed in the 60–69 year age group and those who were categorized as having 10–20% risk of CVD.

**Table 3:** Weighted percentage (95% CI) of Tehranian adults meeting the definition of hypertension and recommended anti-hypertensive medication in those without anti-hypertensive medication (n = 8996) according to the 2017 ACC/AHA guideline and the 2003 JNC7 guideline based on the 5^th^ examination (2012–2015) of TLGS data.

	Hypertension definition				Recommended anti-hypertensive medication		
	Weighted %	2017 ACC/AHA guideline^a^	JNC7 Guideline^b^	Difference (2017 ACC/AHA –JNC7)	2017 ACC/AHA guideline	JNC7 guideline	Difference (2017 ACC/AHA –JNC7)
Overall	89.6	42.7 (42.0–43.7)	12.6 (11.9–13.3)	30.1 (29.0–31.3)	21.9 (21.1–22.7)	19.3 (18.5–20.0)	2.6 (1.4–3.8)
Age groups, (years)							
20–29	23.2	25.1 (23.1–27.0)	4.1 (3.2–5.0)	21.0 (18.9–23.1)	4.5 (3.6–5.5)	4.4 (3.5–5.3)	0.1 (−1.0–1.0)
30–39	28.9	36.7 (35.0–38.8)	7.1 (6.0–8.2)	29.6 (27.3–31.9)	9.5 (8.2–10.8)	9.2 (7.9–10.5)	0.3 (−1.4–2.0)
40–49	19.11	48.8 (46.6–50.9)	12.9 (11.4–14.3)	35.9 (33.3–38.4)	21.7 (19.9–23.4)	20.2 (18.5–22.0)	1.5 (−0.9–4.0)
50–59	14.3	57.2 (55.0–59.7)	20.2 (18.2–22.3)	37.0 (34.0–39.9)	36.5 (34.0–38.9)	32.3 (29.9–34.6)	4.0 (1.0–7.0)
60–69	8.5	59.1 (55.7–62.5)	25.5 (22.5–28.5)	33.6 (30.0–37.1)	59.1 (55.7–62.5)	44.6 (41.2–48.0)	14.5 (1.1–18.0)
70–79	4.09	63.6 (59.1–68.1)	35.5 (31.0–40.0)	28.1 (23.5–32.7)	63.6 (59.1–68.1)	55.0 (50.2–59.5)	8.6 (4.0–13.0)
≥80	1.9	61.1 (51.8–70.0)	34.3 (25.1–43.4)	26.8 (18.3–35.3)	61.1 (51.9–70.2)	57.4 (48.1–66.7)	3.7 (−5.0–13.0)
Gender							
Male	49.8	50.0 (48.5–51.6)	14.9 (13.8–16.0)	35.1 (33.3–36.8)	24.8 (23.6–26.0)	21.2 (20.0–22.4)	3.6 (1.9–5.3)
Female	50.2	35.4 (34.0–36.7)	10.3 (8.2–8.4)	25.1 (23.6–26.5)	19.1 (18.1–20.0)	17.3 (16.4–18.2)	1.8 (0.4–3.1)
10-year risk categories							
<5%	84.4	39.0 (37.9–40.1)	10.1 (0.9–10.7)	28.9 (27.7–30.1)	16.4 (15.6–17.2)	15.1 (14.3–15.8)	1.3 (0.2–2.3)
5%-<10%	6.5	59.0 (55.0–63.1)	18.0 (14.8–21.1)	41.0 (36.2–45.8)	32.5 (28.7–36.3)	29.5 (25.8–33.2)	3.0 (−2.0–8.0)
10%-<20%	5.2	61.7 (57.4–66.0)	25.6 (21.7–29.5)	36.1 (30.9–41.3)	61.7 (57.4–66.0)	40.7 (36.4–45.1)	21.0 (15.5–26.5)
≥20%	3.9	70.2 (66.0–74.5)	40.9 (36.2–45.6)	29.3 (24.5–34.1)	70.2 (66.0–74.5)	63.5 (59.0–68.0)	6.7 (1.9–11.4)
Prevalent CVD	5.0	55.0 (50.3–59.6)	24.5 (20.4–28.5)	30.5 (26.4–34.6)	55.0 (50.3–59.6)	39.1 (34.5–43.7)	15.6 (11.3–20.0)

Definition of Hypertension and Recommended anti-hypertensive medication based on 2017 ACC/AHA and JNC 7 were defined previously in Table 1.

2017 ACC/AHA guideline: 2017 American college of cardiology/American Heart Association guideline for the prevention, Detection, Evaluation, and Management of High Blood pressure in Adults; JNC7 guideline: seventh report of the Joint National Committee on prevention, Detection, and Treatment of High Blood pressure; CVD: Cardiovascular disease.

^a^Un-weighted overall percentage according to the ACC/AHA guideline:(stage 1 + stage 2) of hypertension/(Total participants not on anti-hypertensive medication) = (2712 + 1170)/8996 = 43.15%. The same approach was followed for all reported percentages.

^b^Un-weighted overall percentage according to the JNC7 guideline:(stage 1) of hypertension/(Total participants not on anti-hypertensive medication) = (1170)/8996 = 13%. The same approach was followed for all reported percentages.

After considering adults on anti-hypertensive medication as prevalent hypertensive cases, the updated prevalence of hypertension and the prevalence of adults who recommended anti-hypertensive medication by 2017 ACC/AHA and 2003 JNC7 guideline definitions are illustrated in Sup Table 3. Accordingly, the prevalence of hypertension was 47.1% and 20.4%, using the 2017 ACC/AHA and 2003 JNC7 guidelines definitions, respectively.

**Figure 2:**
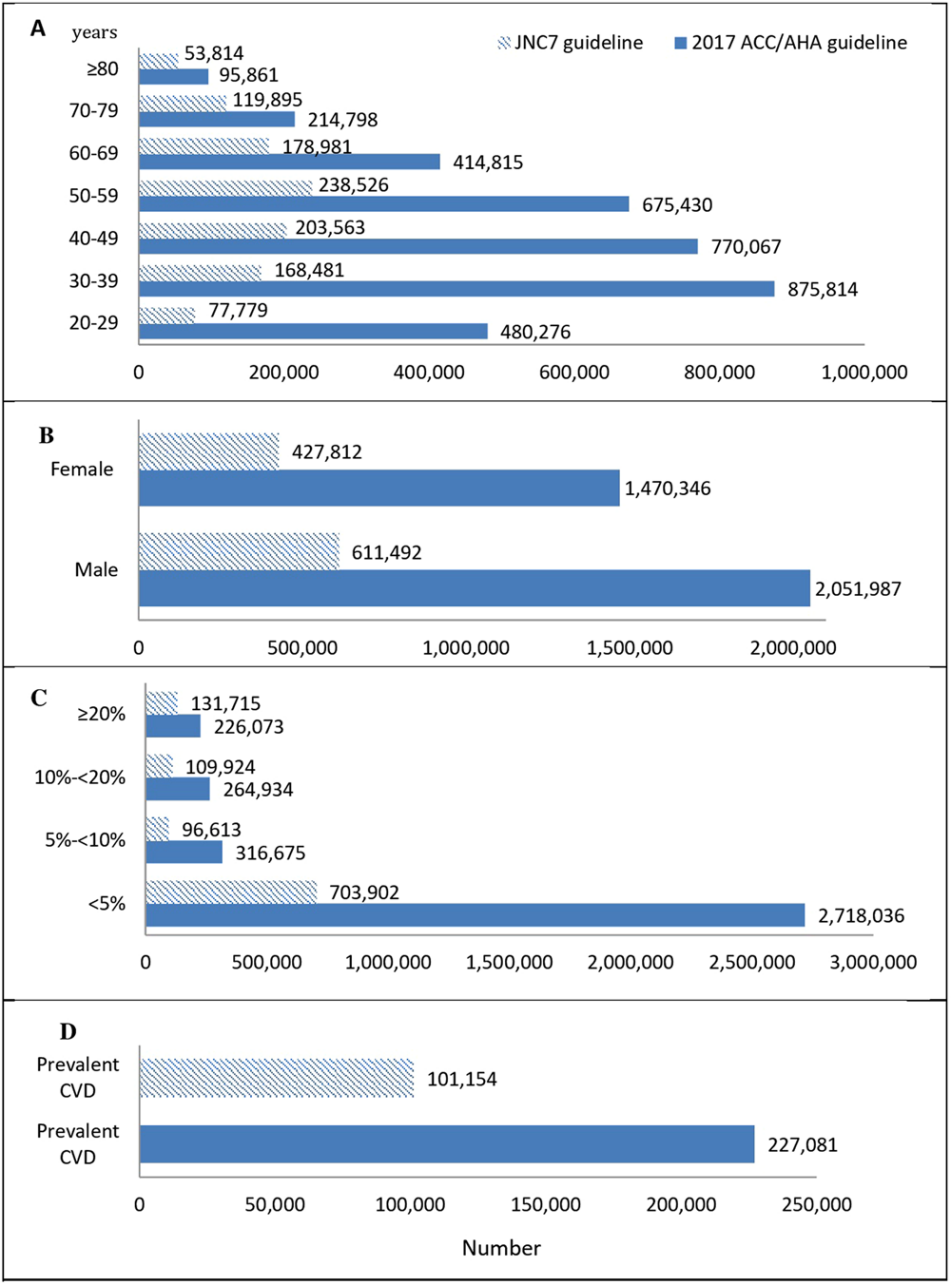
Number of Tehranian adults, meeting the definition of hypertension in those without anti-hypertensive medication according to the 2017 ACC/AHA guideline based on the 5th examination (2012–2015) of TLGS data. 2017 ACC/AHA guideline: 2017 American College of Cardiology/American Heart Association Guideline for the Prevention, Detection, Evaluation, and Management of High Blood Pressure in Adults. JNC7 guideline: The Seventh Report of the Joint National Committee on Prevention, Detection, Evaluation, and Treatment of High Blood Pressure. (**A**) Age categories (years); (**B**) Gender; (**C**) 10-year risk categories (based on ACC/AHA pooled cohort risk equation^16^). (**D**) Among those with prevalent CVD.

According to Fig. 2, of a total 8,257,493 Tehranians, aged ≥20 years 3,525,950 and 1,040,444 Tehranian adults met the definition of hypertension according to the 2017 ACC/AHA and the 2003 JNC7 guidelines, respectively. Overall, of the eligible Tehranian adults who belonged to only the newly diagnosed category (n = 2,485,506), 2,234,197 newly diagnosed participants had 10-year risk <10%, indicating that, about 90% of newly identified cases did not belong to the high-risk category.

**Figure 3:**
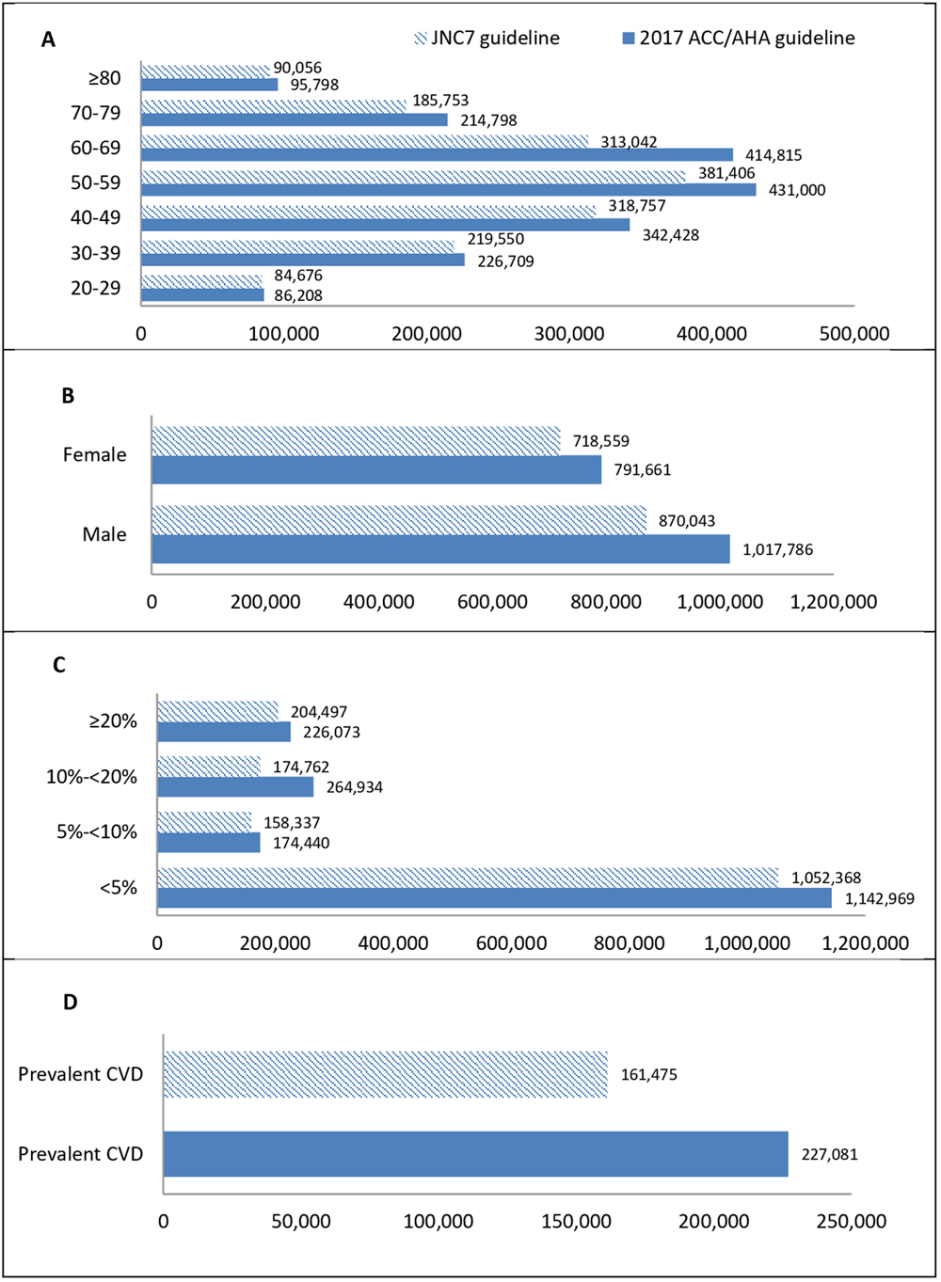
Number of Tehranian adults, meeting the definition of recommended anti-hypertensive medication in those without anti-hypertensive medication according to the 2017 ACC/AHA guideline based on the 5th examination (2012–2015) of TLGS data. 2017 ACC/AHA guideline: 2017 American College of Cardiology/American Heart Association Guideline for the Prevention, Detection, Evaluation, and Management of High Blood Pressure in Adults. JNC7 guideline: The Seventh Report of the Joint National Committee on Prevention, Detection, Evaluation, and Treatment of High Blood Pressure. (**A**) Age categories (years); (**B**) Gender; (**C**) 10-year risk categories (based on ACC/AHA pooled cohort risk equation^16^). (**D**) Among those with prevalent CVD.

With respect to the 2017 ACC/AHA guideline, 1,808,391 Tehranian adults were recommended anti-hypertensive medication vs. 1,593,696 adults using 2003 JNC7 guideline (Fig. 3).

As shown in Table 4, among Tehranian participants taking anti-hypertensive medication, 20% achieved the blood pressure target goal according to the 2017 ACC/AHA guideline, compared to the 42.1% using 2003 JNC7 guideline; a finding consistent over different age groups, both genders and different categories of anti-hypertensive medications.

**Table 4:** Weighted percentage (95% CI) of Tehranian adults taking anti-hypertensive medication (n = 1580) with blood pressure reach to the goal of the 2017 ACC/AHA guideline and 2003 JNC7 guideline treatment goal on the 5th examination (2012–2015) TLGS data.

	Weighted %	Blood pressure reach to the goal according to:		Difference (JNC7–2017 ACC/AHA)
		2017 ACC/AHA guideline	JNC7 guideline	
Overall	10.4	20.0 (14.7–25.3)	42.1 (35.3–48.9)	22.1 (19.0–25.2)
Age groups, (years)				
20–29	23.2	7.0 (−5.7–19.7)	35.0 (18.6–51.4)	28.0 (−9.0–65.4)
30–39	28.9	25.1 (10.9–39.3)	53.4 (35.2–71.6)	28.3 (3.4–53.2)
40–49	19.1	23.2 (16.0–30.4)	45.8 (37.1–54.4)	22.6 (11.3–33.9)
50–59	14.3	22.5 (17.8–27.1)	38.0 (32.3–43.4)	15.5 (8.9–22.0)
60–69	8.5	26.1 (22.2–30.0)	34.0 (29.7–38.2)	7.9 (2.4–13.4)
70–79	4.1	23.3 (19.0–27.6)	27.6 (23.0–32.1)	4.3 (−1.7–10.3)
≥80	1.9	17.6 (10.9–24.3)	18.5 (11.6–25.3)	0.9 (−8.7–10.5)
Gender				
Male	49.3	12.8 (6.5–19.2)	38.7 (30.6–46.8)	25.9 (21.1–30.7)
Female	50.2	27.2 (18.7–35.6)	45.5 (34.6–56.4)	18.3 (14.2–22.4)
Medication				
ACE inhibitor/ARB	53.6	21.2 (12.7–29.8)	50.6 (40.2–61.0)	29.4 (25.1–33.7)
Beta blocker	54.9	20.6 (13.7–27.5)	37.4 (29.6–45.1)	16.8 (12.7–20.9)
Diuretic	10.8	23.2 (14.1–32.3)	38.3 (25.4–51.2)	15.1 (7.8–22.4)
Calcium channel blocker	16.6	13.3 (7.8–18.7)	38.6 (24.0–53.2)	25.3 (19.2–31.3)

Definition of Hypertension and Recommended anti-hypertensive medication based on 2017 ACC/AHA and JNC 7 were defined previously in Table 1.

2017 ACC/AHA guideline: 2017 American college of cardiology/American Heart Association guideline for the prevention, Detection, Evaluation, and Management of High Blood pressure in Adults; JNC7 guideline: seventh report of the Joint National Committee on prevention, Detection, and Treatment of High Blood pressure.

**Figure 4:**
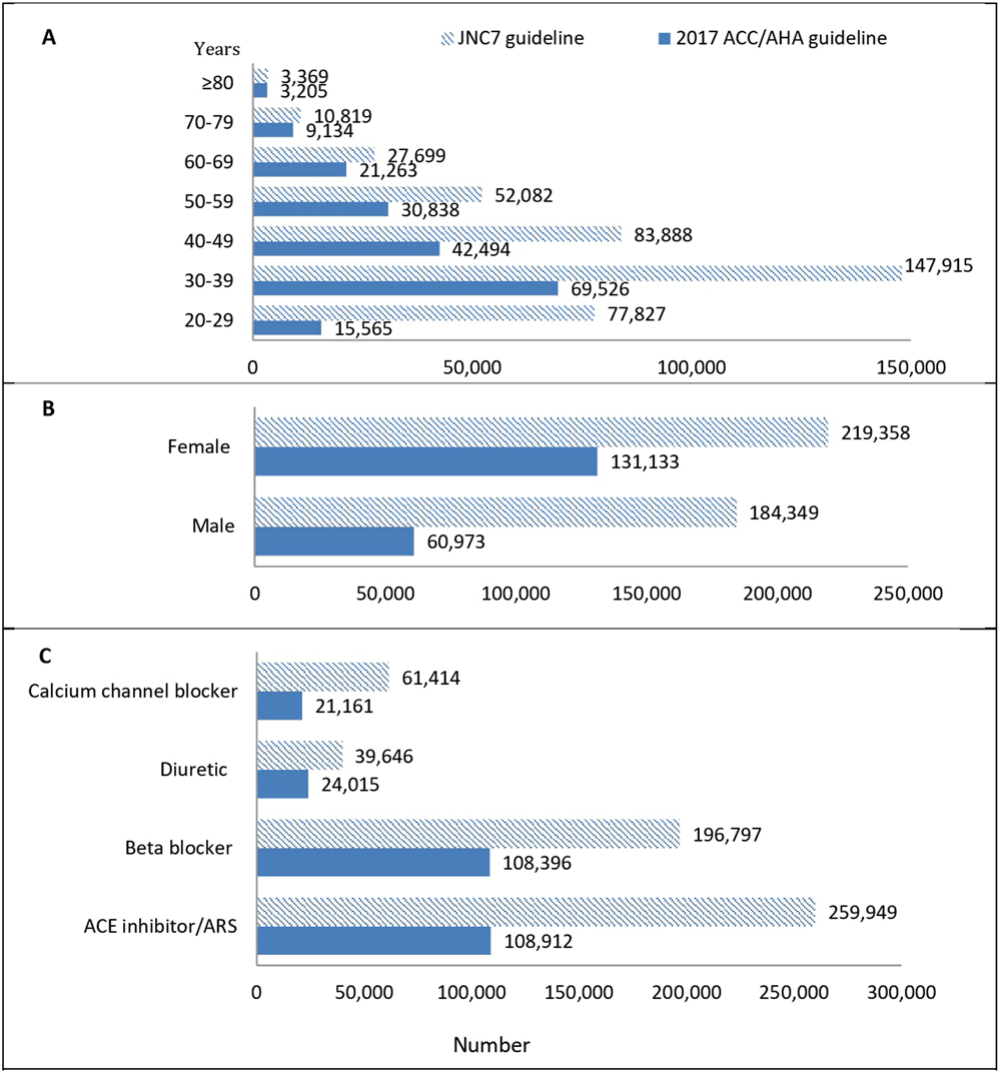
Number of Tehranian adults taking anti-hypertensive medication with blood pressure reach to the goal of the 2017 ACC/AHA guideline and JNC 7 guideline treatment goal of the 5th examination (2012–2015) TLGS data. 2017 ACC/AHA guideline: 2017 American College of Cardiology/American Heart Association Guideline for the Prevention, Detection, Evaluation, and Management of High Blood Pressure in Adults. JNC7 guideline: The Seventh Reportof the Joint National Committee on Prevention, Detection, Evaluation, and Treatment of High Blood Pressure. (**A**) Age categories; (**B**) Gender; (**C**) Medication.

Among Tehranian adults between 2012–2015, 9,215,952 were taking anti-hypertensive medication of whom, 191,692 and 403,511 achieved the blood pressure target goal according to the 2017 ACC/AHA and 2003 JNC7 guidelines, respectively (Fig. 4).

### Comparison of the 2017 ACC/AHA guideline with JNC8 panel member report

Overall, 12.2% of Tehranian adults (1,007,414 individuals), were candidates for anti-hypertensive medication, according to the 2014 JNC8 panel member report guideline vs. 21.9% (1,808,391 individuals) based on 2017 ACC/AHA guideline. The difference between the 2017 ACC/AHA and 2014 JNC8 guidelines in the recommended anti-hypertensive medication was observed in all age groups, in both genders and different 10-year risk categories (Sup Table 4).

As shown in Sup Tables 5 and 6, among Tehranian adults taking anti-hypertensive medication, 53.6% (513,734 individuals) achieved the blood pressure target goal according to the 2014 JNC8 panel member report, compared to the 20% (191,692 individuals) according to the 2017 ACC/AHA guideline.

## Discussion

In the current study we investigated the impact of the 2017 ACC/AHA blood pressure guideline on the prevalence of hypertension, the prevalence of adults recommended antihypertensive medications and those who had achieved blood pressure target goals.

Our results show that the prevalence of hypertension among Tehranian participants not on antihypertensive medication was 42.7% according to the 2017 ACC/AHA blood pressure guideline (47.1% including those treated with anti-hypertensive medication as prevalent hypertensive subjects); this prevalence was almost three times higher than that estimated using 2003 JNC7 criteria, and was over two fold after inclusion of those treated with anti-hypertensive medication as prevalent hypertensive cases. Despite this noticeable increase in the prevalence of hypertension, about 90% these newly identified cases belonged to the low-risk group (10-year risk <10%) and the percentage of adults recommended anti-hypertensive medication; besides non-pharmacological interventions increased only 2.6%, compared to those recommended by the 2003 JNC7 guideline. However, among Tehranian adults taking anti-hypertensive medication, only 20% reached the blood pressure target goals defined by the 2017 ACC/AHA guideline, compared with 42.1% and 53.6% based on the 2003 JNC7 and 2014 JNC8 guidelines, respectively.

The 2017 ACC/AHA guideline has modified the approach to hypertension worldwide in many ways. Data shows that the prevalence of hypertension increased from 31.9 to 45.6% in the USA using the 2017 ACC/AHA guideline, indicating that an additional 31 million individuals now need treatment; of these only 4.2 million are candidates for anti-hypertensive therapy and the rest can be managed by non-pharmacological interventions^12^. This large increase (13.6 vs. 31.5%) was also seen in Peru, a Latin American country^23^. There are three studies that compare the new guideline with previous ones in the East Asia^24–26^; in China, the prevalence of hypertension based on 2017 ACC/AHA guideline was twice as high as that observed, based on the 2010 Chinese guideline (46.4 vs. 23.2%), which defines hypertension by the same thresholds as the 2003 JNC7 guideline^25^.

There are two important aspects that could justify the greater differences in the prevalence of hypertension using 2017 ACC/AHA guideline vs. the 2003 JNC7 guideline among Tehranian populations, compared to those reported in other studies; the first is that the increase in prevalence of hypertension based on 2017 ACC/AHA guideline compared to 2003 JNC7 guideline was more prominent among younger age groups (Table 3). The prevalence of hypertension using 2017 ACC/AHA was 6 times higher than corresponding values using 2003 JNC7 criteria in younger age groups (i.e. 20–29 and 30–39 years), though these differences decreased at older ages; this was also clearly shown in studies conducted among populations from China, Peru and Bangladesh^23,25,27^; for example among a population from Peru it was shown that the prevalence of hypertension based on the 2017 ACC/AHA guideline was almost 5 times higher than those reported in younger age groups by 2003 JNC7, a difference significantly attenuated in older aged participants^23^. These findings demonstrate that the impact of the 2017 ACC/AHA guideline on the prevalence of hypertension is most noticeable in younger populations. In the current study, we enrolled participants, aged ≥20 years, 70% of which were under 50 years old (Table 3), whereas in the studies from Korea and Bangladesh, participants recruited were older^24,27^. Therefore, the marked difference between guidelines could be attributed to the younger population of our study, compared to other studies. The second point that should be considered is the high prevalence and incidence of pre-hypertension among Tehranian populations. Actually in the third national survey of risk factors of non-communicable disease (SuRFNCD) 2011, the prevalence of pre-hypertension was reported to be 39.8% (95% CI: 37.8–41.9)^28^. Moreover, we also reported a high incidence of pre-hypertension, i.e. 593/10000 person-years among an Iranian population^29^. Hence, labeling those with SBP 130–139 mmHg or DBP 80–89 mmHg as hypertensive, instead of pre-hypertensive individuals, using 2017 ACC/AHA guideline, can lead to a dramatic increase in the prevalence of hypertension.

Vaduganathan *et al*. investigated adults with established CVD or at high cardiovascular risk in the USA; based on the 2017 ACC/AHA guideline, 80% of the participants were classified as hypertensive patients at baseline^30^. In our study population, 70.2% of individuals with 10-year risk of CVD ≥20% and 55% of participants with prevalent CVD were classified as the hypertensive category. Many observational studies, as well as a meta-analysis indicated that an increase in blood pressure, even in pre-hypertensive individuals, according to 2003 JNC7 increased the risk of CVD, end-stage renal disease and cerebrovascular events^31–35^. Moreover, previously we also found that, in those with high normal blood pressure (SBP between 130 and 139 mmHg or DBP between 85 and 89 mmHg) the CVD risk increased to >60% among middle-aged Tehranian adults^35^. It is believed that these studies were fundamental for changes made in the 2017 ACC/AHA guideline. The systolic blood pressure intervention trial (SPRINT) evaluated the impact of the 2017 ACC/AHA vs. 2003 JNC7 guidelines on subsequent cardiovascular events; accordingly, in an >3.3-year median follow up, the 2017 ACC/AHA guideline identified a greater number of patients who experienced long-term cardiovascular events^30^.

Another difference of the 2017 ACC/AHA with 2003 JNC7 guidelines was the emphasis on cardiovascular risk. In the 2017 ACC/AHA guideline, the decision for initiating medication is made based on blood pressure in combination with cardiovascular risk and age. Individuals with high cardiovascular risk or age ≥65 years are recommended to initiate antihypertensive therapy at lower levels of blood pressure^9^. Although change in lifestyle is a key factor in the management of hypertension^36^, individuals with prehypertension may benefit from pharmacological interventions at some points, e.g. patients with high cardiovascular risk^37^. The prospective meta-analyses conducted by the Blood Pressure Lowering Treatment Trialists’ indicates that incremental benefits from blood pressure lowering depend on baseline cardiovascular risk^38^. The Heart Outcomes Prevention Evaluation–3 (HOPE3) trial also showed that in participants with approximately 10% 10-year risk, no risk reduction was observed with 6 mmHg decrease in SBP^39^. Compared to the American population, our results showed a minimal increase in the prevalence of adults who are recommended anti-hypertensive medication using the 2017 ACC/AHA guideline (2.6% vs. 1.9%); a great proportion of this increase (21%) was related to individuals with 10-year cardiovascular risk of between 10–20% and those with prevalent CVD (15.59%); corresponding values were 9.9 and 3.7% in the USA, respectively^12^. Hence considering the cardiovascular risk for management of hypertension, affects Iranian populations in a more obvious way, compared to American ones.

Recent trials have shown that hypertensive patients may benefit from more aggressive blood pressure reduction^40–42^. Lewington *et al*. demonstrated a 20 mmHg reduction in SBP was associated with a 40% and 50% decrease in CVD and stroke mortality, respectively^43^. Though the SPRINT trial showed that SBP reduction to <120 mmHg had significantly higher CVD protection, compared to SBP reduction to <140 mmHg, these benefits are obtained at the cost of serious adverse events including syncope, hypotension, acute kidney injuries, and electrolyte abnormalities^42^. A recent systematic review, focusing on clinical trials with a SBP target <130 mmHg, indicated that risk reductions were modestly attenuated compared to those with higher blood pressure target policies, although they were still significant for CVD and stroke^44^. Taking all the cost and benefits into account, the 2017 ACC/AHA guideline determined 130- and 80-mm Hg as blood pressure target goals, which are lower than those recommended by 2003 JNC7. Among Tehranian adults taking anti-hypertensive medication only 20% reached the blood pressure goals according to the 2017 AHA/ACC guideline; corresponding values among treated hypertensive patients in China and the USA were 14.9 and 46.6%, respectively^12,25^.

Prevalence of hypertension was reported to be 25% in Iranian adults, based on the 2003 JNC7 guideline, of whom, only 25% were taking prescribed medication and of those receiving pharmacological treatment, 24% were adequately controlled^13^. Another study using the 2003 JNC7 guideline, which was conducted in a rural area, reported that 41.8% of the Iranian population was hypertensive, 17.6% were treated and 32.1% of those treated had controlled hypertension^14^. Here we showed that the prevalence of hypertension in 2012–2015 in an urban Tehranian population was 12.6%; 19.3% were recommended i.e. anti-hypertensive therapy and 42.1% of those taking medication were well controlled based on JNC7 criteria. Based on the above studies, it can be deduced that we already lacked proper management of hypertension. As mentioned, considering the new guideline the prevalence of hypertension increased to three-fold and the control rate decreased by 50% compared to the 2003 JNC7 guideline. However, most of the newly identified patients were not classified in the high-risk group and pharmacological treatment was not recommended for them, indicating that despite the marked increase in the prevalence of hypertension, only 11% required alterations in the treatment protocol. On the other hand, there is potential psychological harm from disease labeling for the rest^45,46^. Overall the impact of 2017 AHA/ACC guideline on the Tehranian population is often in the vague balance of benefits and harms.

This study is the sixth worldwide^12,23–25,27^ population-based study, to have examined the impact of implementation of the 2017 AHA/ACC guideline on the prevalence of hypertension, among individuals recommended medications and those who have achieved blood pressure target goals. Also, to the best of our knowledge, this study was the first study in the Middle East region, with a high burden of cardiovascular risk factors^7^ that has compared the 2017 AHA/ACC guideline with those of 2003 JNC7 and 2014 JNC 8.

There are however some limitations in our study to note. First, we did not have data of the whole country and our results were limited to Tehran. Second, we used the data of the fifth phase of TLGS cohort, and their awareness and lifestyle may have been modified over these years (cohort effect); our results hence may have underestimated the true prevalence of hypertension and also have overestimated the control rate of hypertension.

The third limitation is that regarding the guidelines for detection of hypertension^47,48^, a raised blood pressure measurement must be confirmed at least at two separate visits to the health care provider’s clinic or office; however this is not applicable in the population-based epidemiological studies^23,27^ and this could overestimate the results due to white coat hypertension.

In conclusion, in this population-based study, the prevalence of hypertension was 42.7% according to the 2017 AHA/ACC blood pressure guideline, compared to 12.6% according to the 2003 JNC7 guideline. Despite the tremendous increase in the prevalence of hypertension in this relatively young population compared to other studies, most of these newly-labeled cases did not belong to the high-risk group and the percentage of adults recommended anti-hypertensive therapy increased about 3%, compared to those recommended by the 2003 JNC7 guideline. Hence, further investigation is needed regarding the possible incremental harm from disease labeling for this large population.

## Supplementary information

Supplementary Tables

## Data Availability

Data are available from the corresponding author on reasonable request.
