# Supplementary material for: Status of Hypertension in Tehran: Potential impact of the ACC/AHA 2017 and JNC7 Guidelines, 2012–2015
Source: Sci Rep. 2019 Apr 23;9:6382. doi: 10.1038/s41598-019-42809-3 (PMC6476880; doi:10.1038/s41598-019-42809-3)
Supplement: Supplementary file 1 — Supplementary Tables [file 41598_2019_42809_MOESM1_ESM.docx]

**Status of Hypertension in Tehran: Potential impact of the ACC/AHA 2017 and JNC7 Guidelines, 2012-2015**

Samaneh Asgari; Msc ^1^, Pegah Khaloo; MD ^2^, Davood Khalili; MD, Ph.D ^1^, Fereidoun Azizi;

MD ^3^, Farzad Hadaegh; MD^1*^

1. Prevention of Metabolic Disorders Research Center, Research Institute for Endocrine Sciences, Shahid Beheshti University of Medical Sciences, Tehran, Iran.
2. Endocrinology and Metabolism Research Center (EMRC), Vali-Asr Hospital, School of Medicine, Tehran University of Medical Sciences, Tehran, Iran.
3. Endocrine Research Center, Research Institute for Endocrine Sciences, Shahid Beheshti University of Medical Sciences, Tehran, Iran

Asgari.S and Khaloo.P contributed equally to this work and are co-first authors.

*Farzad Hadaegh MD.

Prevention of Metabolic Disorders Research Center, Research Institute for Endocrine Sciences, Shahid Beheshti University of Medical Sciences, Tehran, Iran

P.O. Box 19395-4763Tehran, Islamic Republic of Iran

Phone: 98 21 22409301-5

Fax: 98 21 22402463

E-mail: [fzhadaegh@endocrine.ac.ir](mailto:fzhadaegh@endocrine.ac.ir)

| Sup Table 1: Weighted characteristics of participants by the hypertension definition and recommended treatment according to ACC/AHA guideline and 2003 JNC7 guideline based on the 2012-2015 examination of TLGS data. | | | | | | | |
| --- | --- | --- | --- | --- | --- | --- | --- |
|  | Hypertension according to the: | | |  | Recommended treatment by: | | |
|  | 2017 ACC/AHA guideline (n=3882) | JNC7 guideline (n=1170) | Difference  (2017 ACC/AHA –JNC7) (n=2712) |  | 2017 ACC/AHA  guideline (n=2084) | JNC7 guideline  (n=1824) | Difference  (2017 ACC/AHA –JNC7) (n=260) |
| Age, (years) | 42.6(0.05) | 43.0(0.12) | 42.6(0.06) |  | 43.06(0.10) | 43.07(0.10) | 45.1(0.48) |
| Female gender, (%) | 50.2 | 50.2 | 50.2 |  | 50.2 | 50.2 | 43.5 |
| Current smoking, (%) | 24.4 | 25.7 | 24.2 |  | 25.9 | 25.7 | 20.0 |
| SBP, (mm Hg) | 122.4(0.2) | 134.5(0.5) | 117.9(0.2) |  | 128.5(0.5) | 129.2(0.5) | 120.6(0.8) |
| DBP, (mm Hg) | 84.4(0.11) | 92.8(0.25) | 81.7(0.07) |  | 89.1(0.25) | 89.6(0.26) | 81.6(0.38) |
| TC, (mmol/L) | 5.03(0.02) | 5.1(0.04) | 5.01(0.02) |  | 5.1(0.03) | 5.1(0.03) | 5.08(0.08) |
| HDL-C, (mmol/L) | 1.24(0.01) | 1.21(0.01) | 1.24(0.01) |  | 1.21(0.01) | 1.21(0.01) | 1.24(0.03) |
| FPG, (mmol/L) | 5.55(0.02) | 5.7(0.05) | 5.51(0.03) |  | 5.9(0.06) | 6.0(0.06) | 5.3(0.05) |
| eGFR, (ml/min/1.73 m^2^) | 75.1(0.21) | 75.1(0.47) | 75.0(0.24) |  | 73.3(0.43) | 72.8(0.44) | 78.2 (1.70) |
| ACC/AHA Score | 3.3(0.04) | 3.9 (0.09) | 2.9 (0.05) |  | 3.7(0.07) | 3.8(0.08) | 3.5(0.17) |
| Lipid lowering medication, (%) | 5.7 | 5.2 | 6.0 |  | 6.9 | 6.8 | 9.4 |
| Diabetes lowering medication, (%) | 5.8 | 6.5 | 5.7 |  | 12.6 | 13.8 | 0 |
| Diabetes, (%) | 9.1 | 11.1 | 8.5 |  | 19.7 | 21.7 | 0 |
| CKD, (%) | 14.1 | 13.1 | 14.5 |  | 22.0 | 24.7 | 0 |
| Prevalent CVD, (%) | 4.8 | 5.0 | 4.5 |  | 7.8 | 4.8 | 76.3 |
| Mean (SE) for continuous variable and Within category proportion (column %) were reported for categorical variables.  Crude sample size was reported for each blood pressure levels.  SBP: Systolic blood pressure; DBP: Diastolic blood pressure; TC: Total cholesterol; HDL-C: High density lipoprotein cholesterol; FPG: Fasting plasma glucose; eGFR: estimated glomerular filtration rate; CKD: chronic kidney diseases; CVD: Cardiovascular disease. | | | | | | | |

| Sup Table 2: Weighted characteristics of adults taking anti-hypertensive treatment(n=1580) with blood pressure reach to their treatment goal according to ACC/AHA guideline and 2003 JNC7 guideline based on the 2012-2015 examination of TLGS data. | | | |
| --- | --- | --- | --- |
|  | Hypertension according to the: | | |
|  | 2017 ACC/AHA guideline (n=382) | 2003 JNC7 guideline (n=533) | Difference  (2003 JNC7-2017 ACC/AHA)  (n=151) |
| Age, (years) | 47.7(0.32) | 45.5(0.30) | 45.3(0.30) |
| Female gender, (%) | 52 | 43.5 | 44.0 |
| Current smoking, (%) | 25.5 | 24.8 | 25.2 |
| SBP, (mm Hg) | 110.8(0.90) | 117.6(1.15) | 124.7(0.98) |
| DBP, (mm Hg) | 71.8(0.44) | 77.6(0.61) | 82.5(0.58) |
| TC, (mmol/L) | 4.7(0.08) | 4.8(0.11) | 4.9(0.17) |
| HDL-C, (mmol/L) | 1.2 (0.02) | 1.2(0.03) | 1.2(0.03) |
| FPG, (mmol/L) | 6.2(0.18) | 5.8(0.10) | 5.4(0.07) |
| eGFR, (ml/min/1.73 m^2^) | 64.6(1.35) | 70.1(1.30) | 75.4(1.18) |
| ACC/AHA Score | 4.1(0.25) | 3.5(0.14) | 3.0(0.16) |
| Lipid lowering medication, (%) | 30.3 | 29.1 | 25.7 |
| Diabetes lowering medication, (%) | 17.1 | 9.3 | 0 |
| Diabetes, (%) | 20.7 | 11.2 | 0 |
| CKD, (%) | 29.5 | 18.1 | 0 |
| Prevalent CVD, (%) | 21.6 | 23.2 | 21.3 |
| Mean (SE) for continuous variable and Within category proportion (column %) were reported for categorical variables.  Crude sample size was reported for each blood pressure levels.  SBP: Systolic blood pressure; DBP: Diastolic blood pressure; TC: Total cholesterol; HDL-C: High density lipoprotein cholesterol; FPG: Fasting plasma glucose; eGFR: estimated glomerular filtration rate;CKD: chronic kidney dieses ; CVD: Cardiovascular disease. | | | |

| Sup Table 3: Weighted percentage (95% CI) of Tehranian adults meeting the definition of hypertension and recommended anti-hypertensive medication (n=10576) according to the 2017 ACC/AHA guideline and the 2003 JNC7 guideline based on the 5^th^ examination (2012-2015) of TLGS data. | | | | | | | | | | | | |
| --- | --- | --- | --- | --- | --- | --- | --- | --- | --- | --- | --- | --- |
|  | | | | |  | Hypertension definition | | |  | Recommended anti-hypertensive medication | | |
|  | | | | Weighted % |  | 2017 ACC/AHA guideline^1^ | 2003 JNC7  Guideline^2^ | Difference  (2017 ACC/AHA –2003 JNC7) |  | 2017  ACC/AHA guideline | 2003 JNC7  guideline | Difference  (2017 ACC/AHA –2003 JNC7) |
| Overall | | | | 100 |  | 47.1(46.2-48.1) | 20.4(19.8-21.1) | 26.7(25.5-27.9) |  | 27.4(26.6-28.1) | 25.4(24.7-26.1) | 2.0(0.8-3.2) |
| Age groups, (years) | | | |  |  |  |  |  |  |  |  |  |
|  | | | 20-29 | 23.2 |  | 25.3(23.4-27.3) | 4.5(3.5-5.4) | 20.8(18.6-23.0) |  | 4.9(3.9-5.9) | 4.8(3.9-5.8) | 0.1(-1.2-1.5) |
|  | | | 30-39 | 28.9 |  | 37.5(35.5-39.5) | 8.3(7.0-9.5) | 29.2(26.8-31.5) |  | 10.7(9.4-12.1) | 10.3(9.0-11.7) | 0.4(-1.4-2.2) |
|  | | | 40-49 | 19.1 |  | 51.8(49.7-53.8) | 17.9(16.3-19.7) | 33.9(31.3-36.5) |  | 26.2(24.4-28.1) | 24.8(23.0-26.7) | 1.4(1.1-4.0) |
|  | | | 50-59 | 14.3 |  | 65.4(63.2-67.6) | 35.4(33.2-37.6) | 30.0(26.9-33.1) |  | 48.6(46.3-50.1) | 45.0(42.7-47.3) | 3.6(0.4-6.8) |
|  | | | 60-69 | 8.5 |  | 75.0(72.6-77.3) | 54.1(51.4-56.8) | 20.9(17.4-24.4) |  | 75.0(72.6-77.3) | 65.9(63.3-68.5) | 9.1(5.7-12.5) |
|  | | | 70-79 | 4.1 |  | 80.3(77.6-83.0) | 65.5 (61.9-68.2) | 14.8(10.6-18.9) |  | 80.3(77.6-82.9) | 75.4(72.5-78.2) | 4.9(0.9-8.8) |
|  | | | ≥80 | 1.9 |  | 80.6(75.8-85.5) | 67.9(62.1-73.4) | 12.7(5.1-20.3) |  | 80.6(75.8-85.5) | 78.8(73.8-83.8) | 1.8(-5.2-8.9) |
| Gender | | | |  |  |  |  |  |  |  |  |  |
|  | | Male | | 49.8 |  | 40.7(39.6-41.8) | 19.4(18.6-20.2) | 21.3(19.5-23.1) |  | 25.4(24.6-26.2) | 24.3(23.5-25.2) | 1.1(-0.6-2.8) |
|  | | Female | | 50.2 |  | 53.6(52.1-55.1) | 21.5(20.4-22.6) | 32.1(30.4-33.7) |  | 29.3(28.2-30.5) | 26.6(25.4-27.7) | 2.7(1.1-4.3) |
| 10-year risk categories | | | | |  |  |  |  |  |  |  |  |
|  | <5% | | | 84.2 |  | 42.6(41.6-43.6) | 16.3(16.6-17.0) | 26.3(25.0-27.6) |  | 21.0(20.2-21.7) | 20.0(19.4-20.8) | 1.0(-0.2-2.2) |
|  | 5%-<10% | | | 6.3 |  | 63.1(59.3-66.8) | 25.4(22.0-28.8) | 37.7(32.7-42.7) |  | 38.2(34.4-42.0) | 35.8(32.0-39.5) | 2.4(-2.8-7.6) |
|  | 10%-<20% | | | 4.9 |  | 70.2(66.6-73.9) | 41.3(37.3-45.2) | 28.9(23.6-34.2) |  | 70.2(66.6-74.0) | 53.5(49.4-57.5) | 16.7(11.3-22.0) |
|  | ≥20% | | | 4.6 |  | 83.8(81.2-86.4) | 67.3(64.0-70.1) | 16.5(12.2-20.8) |  | 83.8(81.1-86.4) | 80.0(77.2-82.8) | 3.8(-0.1-7.7) |
| Prevalent CVD | | | | 6.9 |  | 76.9(74.1-79.7) | 60.8(57.6-64.0) | 16.1(12.1-20.1) |  | 76.9(74.1-79.7) | 67.6(64.4-70.7) | 9.3(5.4-13.2) |
| Definition of Hypertension and Recommended anti-hypertensive medication based on 2017 ACC/AHA and 2003 JNC 7 were defined previously in Table 1.  2017 ACC/AHA guideline: 2017 American college of cardiology/American Heart Association guideline for the prevention, Detection, Evaluation, and Management of High Blood pressure in Adults; JNC7 guideline: seventh report of the Joint National Committee on prevention, Detection, and Treatment of High Blood pressure; CVD: Cardiovascular disease.  ^1^ Un-weighted overall percentage according to the ACC/AHA guideline: (stage 1 +stage 2+treated participants) of hypertension/ (Total population) = (2712+1170+1580)/ 10576=51.6%. The same approach was followed for all reported percentages.  ^2^ Un-weighted overall percentage according to the JNC7 guideline: (stage 1+treated participants) of hypertension/ (Total participants) = (1170+1580)/ 10576=26.0%. The same approach was followed for all reported percentages. | | | | | | | | | | | | |

| Sup Table 4: Weighted percentage (95% CI) and Number of Tehranian adults meeting the definition of recommended anti-hypertensive medication according to the 2017 ACC/AHA guideline and the 2014 JNC8 guideline based on the 5^th^ examination (2012-2015) of TLGS data. | | | | | | | | |
| --- | --- | --- | --- | --- | --- | --- | --- | --- |
|  | | weighted percentage (95% CI) | | |  | Number of Tehranian adults, in millions | | |
|  | | 2017 ACC/AHA guideline | 2014 JNC8 guideline | Difference  (2017 ACC/AHA –2014 JNC8) |  | 2017 ACC/AHA guideline | 2014 JNC8 guideline | Difference  (2017 ACC/AHA –2014 JNC8) |
| Overall | | 21.9(21.1-22.7) | 12.2(11.5-12.8) | 9.7(8.6-10.8) |  | 1,808,391 | 1,007,414 | 800,977 |
| Age groups, (years) | |  |  |  |  |  |  |  |
|  | 20-29 | 4.5(3.6-5.5) | 4.1(3.2-5.0) | 0.4(-0.8-1.7) |  | 86,208 | 77,779 | 8,429 |
|  | 30-39 | 9.5(8.2-10.8) | 7.1(6.0-8.2) | 2.4(0.8-4.08) |  | 226,709 | 168,481 | 58,229 |
|  | 40-49 | 21.7(19.9-23.4) | 12.9(11.4-14.3) | 8.8(6.6-11.0) |  | 342,428 | 203,563 | 138,865 |
|  | 50-59 | 36.5(34.0-38.9) | 20.2(18.2-22.3) | 16.3(13.4-19.2) |  | 431,000 | 238,526 | 192,474 |
|  | 60-69 | 59.1(55.7-62.5) | 22.0(19.1-24.8) | 37.1(33.6-40.5) |  | 414,815 | 154,415 | 260,400 |
|  | 70-79 | 63.6(59.1-68.1) | 33.9(29.5-38.2) | 29.7(25.1-34.2) |  | 214,798 | 114,491 | 100,306 |
|  | ≥80 | 61.1(51.9-70.2) | 32.0(23.0-41.0) | 29.1(20.7-37.4) |  | 95,798 | 50,205 | 45,593 |
| Gender | |  |  |  |  |  |  |  |
|  | Male | 24.8(23.6-26.0) | 14.4(13.3-15.4) | 10.4(8.8-12.0) |  | 1,017,786 | 590,972 | 426,813 |
|  | Female | 19.1(18.1-20.0) | 10.0(9.2-10.8) | 9.1(7.8-10.3) |  | 791,661 | 415,352 | 376,309 |
| 10-year risk categories | |  |  |  |  |  |  |  |
|  | <5% | 16.4(15.6-17.2) | 9.9(9.2-10.1) | 6.5(5.5-7.5) |  | 1,142,969 | 689,963 | 453,006 |
|  | 5%-<10% | 32.5(28.7-36.3) | 18.0(14.8-21.1) | 14.5(9.8-19.2) |  | 174,440 | 96,613 | 77,827 |
|  | 10%-<20% | 61.7(57.4-66.0) | 23.5(19.7-27.3) | 38.2(33.1-43.3) |  | 264,934 | 100,907 | 164,027 |
|  | ≥20% | 70.2(66.0-74.5) | 37.0(32.4-41.6) | 33.2(28.4-38.0) |  | 226,073 | 119,156 | 106,918 |
| Prevalent CVD | | 55.0(50.3-59.6) | 23.5(19.5-27.5) | 23.0(18.8-27.2) |  | 227,081 | 97,026 | 94,961 |
| Definition of Recommended anti-hypertensive medication based on 2017 ACC/AHA and 2014 JNC 8 were defined previously in Table 1.2017 ACC/AHA guideline: 2017 American college of cardiology/American Heart Association guideline for the prevention, Detection, Evaluation, and Management of High Blood pressure in Adults; JNC8 guideline: eighth report of the Joint National Committee on prevention, Detection, and Treatment of High Blood pressure; CVD: Cardiovascular disease. | | | | | | | | |

| Sup Table 5: percentage (95% CI) of Tehranian adults taking anti-hypertensive medication (n=1580) with blood pressure reach to the 2017 ACC/AHA guideline and 2014 JNC8 guideline treatment goal of the 5th examination (2012-2015) TLGS data. | | | | | |
| --- | --- | --- | --- | --- | --- |
|  | | Blood pressure reaches to goal, according to: | |  | Difference (2017  ACC/AHA –2014 JNC8) |
|  | | 2017 ACC/AHA guideline | 2014 JNC8 guideline |  |  |
| Overall | | 20.0(14.7-25.3) | 53.6(47.2-60.0) |  | 33.6(30.4-36.7) |
| Age groups, (years) | |  |  |  |  |
|  | 20-29 | 7.0(-5.7-19.7) | 35.0(18.6-51.4) |  | 28.0(-9.5-65.5) |
|  | 30-39 | 25.1(10.9-39.3) | 68.4(51.9-84.8) |  | 43.3(19.3-67.3) |
|  | 40-49 | 23.2(16.0-30.4) | 53.7(45.0-62.4) |  | 30.5(19.2-41.8) |
|  | 50-59 | 22.5(17.8-27.1) | 54.0(48.3-59.7) |  | 31.5(24.8-38.2) |
|  | 60-69 | 26.1(22.2-30.0) | 58.5(54.0-62.9) |  | 32.4(26.8-38.0) |
|  | 70-79 | 23.3(19.0-27.6) | 51.7(46.7-56.8) |  | 28.4(22.0-34.8) |
|  | ≥80 | 17.6(10.9-24.3) | 34.0(25.6-42.4) |  | 16.4(5.6-27.1) |
| Gender | |  |  |  |  |
|  | Male | 12.8(6.5-19.2) | 56.1(45.1-67.1) |  | 43.3(38.4-48.2) |
|  | Female | 27.2(18.7-35.6) | 51.1(44.6-57.6) |  | 23.9(-19.8-28.0) |
| Medication | |  |  |  |  |
|  | ACE inhibitor | 21.2(12.7-29.8) | 67.7(58.7-76.8) |  | 46.5(42.3-50.6) |
|  | Beta blocker | 20.6(13.7-27.5) | 46.0(39.0-52.9) |  | 25.4(21.2-29.6) |
|  | Diuretic | 23.2(14.1-32.3) | 64.1(49.2-79.0) |  | 40.9(33.7-48.1) |
|  | Calcium channel blocker | 13.3(7.8-18.7) | 55.7(39.8-71.6) |  | 42.4(36.3-48.5) |
| Definition of Hypertension and Recommended anti-hypertensive medication based on 2017 ACC/AHA and 2014 JNC 8 were defined previously in Table 1.  2017 ACC/AHA guideline: 2017 American college of cardiology/American Heart Association guideline for the prevention, Detection, Evaluation, and Management of High Blood pressure in Adults; JNC8 guideline: eighth report of the Joint National Committee on prevention, Detection, and Treatment of High Blood pressure | | | | | |

| Sup Table 6: Number of Tehranian adults taking anti-hypertensive medication with blood pressure reach to the 2017 ACC/AHA guideline and 2014 JNC 8 guideline treatment goal on the 5th examination (2012-2015) TLGS data. | | | | | |
| --- | --- | --- | --- | --- | --- |
|  | | Blood pressure reach to the goal according to: | |  | Difference  (2014 JNC8-2017 ACC/AHA ) |
|  | | 2017 ACC/AHA guideline | 2014 JNC8 guideline |  |  |
| Overall | | 191,692 | 513,734 |  | 322,042 |
| Age groups, (years) | |  |  |  |  |
|  | 20-29 | 15,565 | 77,827 |  | 62,262 |
|  | 30-39 | 69,526 | 189,465 |  | 119,939 |
|  | 40-49 | 42,494 | 98,358 |  | 55,864 |
|  | 50-59 | 30,838 | 74,012 |  | 43,174 |
|  | 60-69 | 21,263 | 47,659 |  | 26,396 |
|  | 70-79 | 9,134 | 20,267 |  | 11,133 |
|  | ≥80 | 3,205 | 6,191 |  | 2,986 |
| Gender | |  |  |  |  |
|  | Male | 60,973 | 267,235 |  | 206,261 |
|  | Female | 131,133 | 246,356 |  | 115,223 |
| Medication | |  |  |  |  |
|  | ACE inhibitor | 108,912 | 347,798 |  | 238,886 |
|  | Beta blocker | 108,396 | 242,049 |  | 133,653 |
|  | Diuretic | 24,015 | 66,352 |  | 42,337 |
|  | Calcium channel blocker | 21,161 | 88,621 |  | 67,460 |
| Definition of Hypertension and Recommended anti-hypertensive medication based on 2017 ACC/AHA and 2014 JNC 8 were defined previously in Table 1.  2017 ACC/AHA guideline: 2017 American college of cardiology/American Heart Association guideline for the prevention, Detection, Evaluation, and Management of High Blood pressure in Adults; JNC8 guideline: eighth report of the Joint National Committee on prevention, Detection, and Treatment of High Blood pressure | | | | | |
